# A Case of Puerpural Convulsions—Tedious Labor, Etc., Etc.

**Published:** 1870-11

**Authors:** N. S. Davis

**Affiliations:** Professor Practical Medicine


ARTICLE XXXVII.

### A CASE OF PUERPURAL CONVULSIONS—TEDIOUS LABOR, ETC., ETC.

BY N. S. DAVIS, M.D., Professor Practical Medicine.

Mrs. M., a lady aged about 30 years; below the medium size; nervous temperament; was married little more than seven years since.

Previous to marriage she had suffered some from dysmenorrhœa, and, soon after marriage, there was added slight leucorrhœal discharge, with a sense of pressure behind the pubes; a disposition to urinate more frequently than natural, and moderate constipation. The urine, however, was natural in quality and quantity, except occasionally, when she had paroxysms of palpitation and sleeplessness, when it would become abundant and limpid. After she had been married three or four years, and not becoming pregnant, while the foregoing symptoms were slowly increasing from month to month, she sought medical advice. From the commencement of her married life, she had been anxious to become a mother, and felt much disappointed that pregnancy had been so long delayed. On making a vaginal examination, I found the neck and os uteri small, hard, and turned slightly backward, while the anterior wall of the body of the uterus rested against the bas fund of the bladder, and the anterior wall. of the vagina appeared unusually short. In other words, the neck and os were small, with partial anteversion.

These conditions explained the pressure behind the pubes, and the desire to urinate often, but were hardly sufficient to explain the entire exemption from pregnancy. The general health and nutrition were good, although subject to periods of nervousness, more especially at her monthly sickness, which was very regular in its recurrence. About three years since, while I was absent from the city, she was under the care of Prof. W. H. Byford, and she spent the following winter in Florida, with some friends. With the exception of some nervous paroxysms, her general health remained good, and her menstruation regular.

Little more than a year since, she again consulted me, with direct reference to her sterility. Examination revealed the uterus and vagina in the same conditions as previously described, except a little more tenderness on pressure against the anterior and lower part of the uterus, sending some pain in the direction of the left ovarium. The opening through the os and uterine neck was not contracted, and the amount of menstrual fluid was sufficient. Neither was there any discharge from the os indicating inflammation in the uterine cavity. There was, on the outer margin of the os, a slight abrasion, with undue redness, to which I applied a solution of nitrate of silver several times, and it entirely disappeared. She also used, once daily, a solution of sulphate of zinc, and morphia—(10 grains of the first and 1 grain of the second, in 3 ounces of water)—as a vaginal wash. Every effort to correct the partial anteversion was fruitless, apparently, from the shortness of the anterior part of the vaginal walls. Thinking it might modify favorably the nervous system of organic life, I gave the patient, internally, the liquor arsenicalis, or Fowler's solution, in doses of 8 drops, at each mealtime, well diluted with water. She continued this course of treatment, with occasional interruptions of a few days at a time, for about three months, when the failure in the appearance of the menses at the proper time, with a characteristic nausea, created suspicions of pregnancy. Time proved the correctness of these suspicions. During the first four months, she suffered almost constant nausea, with more annoyance from a frequent desire to urinate than previously; but, otherwise, her health continued good through the whole period of pregnancy. After the supposed full completion of the period of pregnancy, I was called, hastily, on the morning of the 21st of September, 1870, the messenger stating that the lady was in convulsions. On arrival, she had just recovered from a severe paroxysm of general convulsions.

The pulse was only slightly accelerated; skin natural; pupils of eyes natural; mind clear; and urinary secretion apparently free. She had complained of headache several days, and the pain had been severe during the preceding night. There were no labor pains, and an examination, per vagina, showed a full development of the neck, and os uteri, but no dilatation whatever. Indeed the finger could detect no opening, the whole uterine surface within the vagina appearing smooth and firm, with the head resting against it, without any perceptible amount of intervening fluid.

Hoping to relieve the headache, and prevent the recurrence of convulsions until the labor-pains should come on, I directed a solution of bromide and iodide of potassia (15 grs. of the first and 5 grs. of the second) to be given every two hours. Saw her again about 5 o'clock P.M., the convulsions having recurred with considerable severity, three or four times during the afternoon.

Her face was now flushed; head hot and painful; pulse and respiration moderately accelerated, but no labor-pains, and no dilatation of the os uteri.

I opened a vein in her arm and took about sixteen ounces of blood, which gave immediate relief to the headache. This was followed by a powder of calomel, 5 grs., leptandrin, 3 grs., and bi-carb. soda, 5 grs., to move the bowels, and 15 drops of tincture of Calabar bean, every two hours, in the place of the bromide and iodide of potassa. She passed the night comfortably, the bowels moving two or three times, the kidneys acting about natural, and no recurrence of the convulsions or headache. There were slight pains in the lower part of the back, but no perceptible change in the condition of the os uteri. During the day, the pain in the back increased and became regularly paroxysmal, recurring every twenty or thirty minutes, but not severe. The head of the child was pressed more firmly downward, and the walls of the uterus, as felt through the vagina, were thinner, but still no opening of the os. During the first part of the night, the labor-pains steadily increased in force and frequency, and her general condition remained good.

The head of the child had fairly entered the superior straight of the pelvis, the uterine walls felt thin and tense from the pressure of the head, but no indication of liquor amnii, nor any dilatation of the os. A very careful examination with the finger, during the latter part of the night, finally identified the os uteri with an opening hardly large enough to admit a quill. By working, in the absence of the pains, when the parts were more relaxed, I succeeded in insinuating my finger into the opening. The edges of the os were very thin, but firm and with each pain, contracted like a sphincter muscle. By careful and persistent efforts with the finger, for more than two hours, the rigidity of the os was finally overcome, and the labor appeared to progress well, though slowly. By 10 o'clock A.M., of the 22d, the head of the child occupied the cavity of the pelvis, but not low enough to rest on the perineum, and the mouth of the womb had not fully receded over the head. Then the labor became stationery; and though the pains continued regular, and with a fair degree of force, hardly any progress was made for three hours. The patient becoming more restless and wearied with the tediousness of the labor, I succeeded in carefully adjusting the forceps and completing the delivery; but it required a full hour, making the traction with the natural pains, and finally increasing the force to the full extent of my ability. The child was partially asphyxiated, but soon recovered its respiration. Laceration of the perineum was wholly avoided, and mother and child progressed favorably, and are now in good health. I have thought this case worth recording, as having some bearing on the following questions: 1st. Did the use of the arsenical preparation have anything to do with the supervention of pregnancy after more than six years of barrenness? 2d. Did the use of the tincture of Calibar bean aid materially in preventing the continuance of the convulsions? That the *bleeding* was of essential service I have no doubt. The immediate relief to the headache and fever were so marked as to leave no doubt of its beneficial influence. I have had opportunity to use the Calibar bean in only two cases of puerperal convulsions—In both the patient recovered; but how much influence for good was attributable to this remedy I am unable to say—I only call attention to the facts.

